# Seasonal variations of patients presenting dyspnea to emergency departments in Europe: Results from the EURODEM Study

**DOI:** 10.3906/sag-2002-221

**Published:** 2020-12-17

**Authors:** Mehmet Akif KARAMERCAN, Zerrin Defne DÜNDAR, Mehmet ERGİN, Oene VAN MEER, Richard BODY, Veli-Pekka HARJOLA, Franck VERSCHUREN, Michael CHRIST, Adela GOLEA, Jean CAPSEC, Cinzia BARLETTA, Luis GARCIA-CASTRILLO, Yusuf Ali ALTUNCI, Yavuz KATIRCI, Anne-Maree KELLY, Said LARIBI

**Affiliations:** 1 Department of Emergency Medicine, Faculty of Medicine, Gazi University, Ankara Turkey; 2 Department of Emergency Medicine, Meram Faculty of Medicine, Necmettin Erbakan University, Konya Turkey; 3 Department of Emergency Medicine, Faculty of Medicine, Ankara Yıldırım Beyazıt University, Ankara Turkey; 4 Department of Emergency Medicine, Leiden University Medical Center, Leiden Netherlands; 5 Department of Emergency Medicine, Central Manchester University Hospitals NHS Foundation Trust, Manchester UK; 6 Department of Cardiovascular Sciences, The University of Manchester, Manchester UK; 7 Department of Emergency Medicine, Faculty of Medicine, University of Helsinki, Helsinki Finland; 8 Department of Emergency Medicine and Services, Helsinki University Hospital, Helsink Finland; 9 Department of Acute Medicine, Université Catholique de Louvain, Cliniques Universitaires Saint-Luc, Brussels Belgium; 10 Department of Emergency Medicine, Luzerner Kantonsspital, Luzern Switzerland; 11 Department of Emergency Medicine, County Emergency Hospital Cluj-Napoca, University of Medicine and Pharmacy, Cluj-Napoca Romania; 12 Department of Public Health, Tours University Hospital, Tours France; 13 Department of Emergency Medicine, Santa Eugenio Hospital, Rome Italy; 14 Servicio Urgencias Hospital Marqués de Valdecilla, Santander Spain; 15 Department of Emergency Medicine, Faculty of Medicine Hospital, Ege University, İzmir Turkey; 16 Department of Emergency Medicine, Ankara Education and Research Hospital, Ankara Turkey; 17 Joseph Epstein Centre for Emergency Medicine Research, Western Health, Melbourne Australia; 18 Department of Medicine, Melbourne Medical School – Western Precinct, The University of Melbourne, Melbourne, Australia; 19 Department of Emergency Medicine, Faculty of Medicine, Tours University, Tours France

**Keywords:** Dyspnea, seasonal variations, emergency department, older patient

## Abstract

**Background/aim:**

To describe seasonal variations in epidemiology, management, and short-term outcomes of patients in Europe presenting to an emergency department (ED) with a main complaint of dyspnea.

**Materials and methods:**

An observational prospective cohort study was performed in 66 European EDs which included consecutive patients presenting to EDs with dyspnea as the main complaint during 3 72-h study periods. Data were collected on demographics, comorbidities, chronic treatment, prehospital treatment, mode of arrival of patient to ED, clinical signs at admission, treatment in the ED, ED diagnosis, discharge from ED, and in-hospital outcome.

**Results:**

The study included 2524 patients with a median age of 69 (53–80) years old. Of the patients presented, 991 (39.3%) were in autumn, 849 (33.6%) were in spring, and 48 (27.1%) were in winter. The winter population was significantly older (P < 0.001) and had a lower rate of ambulance arrival to ED (P < 0.001). In the winter period, there was a higher rate for lower respiratory tract infection (35.1%), and patients were more hypertensive, more hypoxic, and more hyper/hypothermic compared to other seasons. The ED mortality was about 1% and, in hospital, mortality for admitted patients was 7.4%.

**Conclusion:**

The analytic method and the outcome of this study may help to guide the allocation of ED resources more efficiently and to recommend seasonal ED management protocols based on the seasonal trend of dyspneic patients.

## 1. Introduction

Shortness of breath, medically known as dyspnea, is a common presenting symptom in emergency departments (ED) and may sometimes be a diagnostic challenge. Dyspnea roughly accounts for 3%–6% of all adult ED presentations and represents up to 50% of patients admitted to acute tertiary care hospitals [1–3]. It has a wide range of possible causes including acute hearth failure, pulmonary diseases, chronic obstructive pulmonary disease (COPD), asthma, pulmonary embolism, pneumonia, etc., metabolic or mixed with a significant predominance of respiratory causes for dyspnea (more than 50% of all cases) [3–5]. Most of these have known seasonal variations and, especially in terms of pulmonary causes, they are often overstretched during winter due to air pollution and an increase in infections [6,7]. However, most of these data are derived from disease-specific studies and/or carried out on patients of pulmonology/cardiology clinics. By nature, these studies exclude significant proportion of patients, i.e. those with mixed diseases. These patients may require different therapeutic approaches for optimal outcomes, which is of great importance in ED patients. On the other hand, the utilization and overcrowding of EDs is an increasing worldwide problem that is clearly associated with an increased risk of morbidity and mortality, especially for patients with time-sensitive conditions such as dyspneic patients [8]. In studies focusing on ED patients, those who presented with dyspnea as a major complaint, different size and follow-up duration is currently used. Less attention has been paid to the potential effects of seasonal changes in regard to occurrence and ED management of these patients. As little is known about dyspneic patients presenting to EDs in Europe, determination of the seasonal variations of these patients, outcomes, and regional differences could help to allocate ED resources efficiently, not only for diagnostic and treatment resources but also for staffing levels and referrals to specific wards. Therefore, the aim of this study is to describe seasonal variations in the epidemiology, management, and short-term outcomes of patients presenting to EDs with dyspnea as a main complaint.

## 2. Materials and methods

### 2.1. Study design

The methodology of this international, multicenter, prospective, observational, interrupted time series cohort study has been published previously [5]. In summary, the European Dyspnea in Emergency Departments (EURODEM) conducted a study at 3 time points in 66 EDs in Europe of consecutive adult patients presenting to ED with dyspnea as the main complaint. This planned substudy includes patients from the EURODEM study and mainly evaluates epidemiology and outcomes of patients presenting to the ED with dyspnea as a main complaint over 3 different seasons. The study sample was generated with the collection of the consecutive patients data over 3 72-h periods in February, May, and October of 2014 (winter, spring, and autumn) and included demographics, mode of ED arrival, comorbidities, vital and clinical signs at admission, treatment in ED, ED diagnosis, disposition from ED, and in-hospital outcome for the hospitalized patients.

### 2.2. Patient population and data collection

Eligible patients were consecutive adult patients presenting to the ED with dyspnea as their main symptom with 30-day follow-up via phone interview. The research was conducted in accordance with the Declaration of Helsinki. Ethical approvals were obtained for all sites according to local requirements. In most institutions, patient consent for data collection was obtained. Consent could be oral (in person or by phone), written, by email, or short messaging service according to local requirements.

A specifically designed data collection form was developed by the steering committee and given to those selected and invited to participate based on their interest in emergency medicine research. The data form was validated with a pilot study on a small sample of cases. Local data collectors were instructed that dyspnea was considered a main symptom if it was listed as a symptom by the triage nurse. Data were collected onto the validated data form by local investigators.

### 2.3. Outcome measures

Proportions of total ED cases, ED diagnoses distribution, ED disposition, and short-term outcome were compared by season of inclusion.

### 2.4. Statistical analysis

The normality analyses of the data were carried out using the Kolmogorov–Smirnov test and histograms. Qualitative variables were expressed as frequency (percentage), and the quantitative variables were expressed as the median (first and third quartile). Comparisons of qualitative variables were performed using a chi-square test. The intergroup differences between the quantitative variables were evaluated using the Kruskal–Wallis test, the Mann–Whitney U-test (with Bonferroni correction), and the one-way ANOVA test with Tukey HSD as appropriate. A formal sample size calculation was not performed in this descriptive study. A two-sided P-value less than 0.05 was considered statistically significant. Statistical analysis was carried out using SAS version 9.1 Software (SAS Institute, Cary, NC, USA).

## 3. Results

### 3.1. Demographic properties of patient population

Sixty-six EDs in Europe contributed with 684 (27.1%) patients recruited in winter (February), 849 (33.6%) patients in spring (May), and 991 (39.3%) patients in autumn (October). The most common comorbidities were hypertension, COPD, dyslipidemia, chronic heart failure, and diabetes mellitus. Patient characteristics are summarized in Table 1.

**Table 1:** Patient characteristics.

	Winter (February) (N = 684)		Spring (May) (N = 849)		Autumn (October) (N = 991)		
	N (%)	Missing Data	N (%)	Missing Data	N (%)	Missing Data	P-value
Age, years *	72 (58-81)	5	68 (53-80)	6	68 (48-80)	11	<0.001
Sex, male	342 (50.4)	5	427 (50.5)	4	455 (46.2)	7	0.119
Duration of symptoms, days *	3 (2–7)	128	2 (1–7)	131	2 (1–6)	174	0.627
Arrival mode, ambulance	293 (43.3)	7	438 (52.0)	7	520 (53.3)	15	<0.001
ED diagnoses LRTI Heart failure COPD exacerbation Asthma Other	240 (35.1) 179 (26.2) 150 (21.9) 46 (6.7) 205 (30.0)		235 (27.7) 183 (21.6) 154 (18.1) 86 (10.1) 288 (33.9)		298 (30.1) 185 (18.7) 162 (16.4) 89 (9.0) 358 (36.1)		
Comorbidities Hypertension COPD Dyslipidemia Chronic heart failure Diabetes mellitus Smoker Ischemic heart disease Asthma Atrial fibrillation/flutter Obesity Renal impairment Active malignancy	343 (53.1) 210 (33.1) 182 (29.6) 182 (28.8) 179 (27.5) 146 (23.5) 174 (27.7) 105 (16.2) 120 (18.6) 137 (21.8) 90 (13.8) 57 (8.9)	38 50 68 53 34 63 55 37 39 54 32 42	374 (46.6) 256 (31.7) 189 (24.0) 185 (23.4) 181 (22.5) 186 (25.4) 175 (22.5) 143 (17.5) 134 (16.7) 138 (17.8) 106 (13.1) 80 (10.0)	46 42 62 58 43 117 72 33 45 75 39 47	418 (44.7) 290 (30.9) 170 (18.9) 212 (23.0) 189 (20.2) 214 (25.0) 152 (16.9) 184 (19.7) 150 (16.0) 107 (11.9) 101 (10.9) 89 (9.7)	55 51 89 68 53 134 91 55 54 94 62 69	0.003 0.637 <0.001 0.018 0.003 0.703 <0.001 0.198 0.388 <0.001 0.172 0.773
*Data are presented as median (Q1–Q3) ED: Emergency Department; COPD: Chronic Obstructive Pulmonary Disease.							

### 3.2. Clinical management and outcomes of the patient population

Of all of the patients, 116 (4.7%) had systolic blood pressure <100 mmHg, 224 (9.1%) had pulse rate >120 beats/min, and 216 (10.5%) had a respiratory rate of >30 breaths/min on admission to ED. The most common clinical signs on admission to ED were rales (44.7%), wheezing (28.9), and rhonchi (25.9%) (Table 2). The most common treatment modalities used in ED were supplemental oxygen (61.0%), inhaled bronchodilator (37.9%), antibiotics (27.7%), and intravenous diuretics (22.5). All 3 treatment modalities were higher during the winter period (Table 3). Of all patients, 921 (39.4%) were discharged from ED to their homes, 1275 (53.2%) were admitted to a ward, and 180 (7.5%) were admitted to an intensive care unit.

**Table 2:** Clinical signs of patients on admission to emergency department.

	Winter (February) (N = 684)		Spring (May) (N = 849)		Autumn (October) (N = 991)		
	N (%)	Missing Data	N (%)	Missing Data	N (%)	Missing Data	P-value
Vital signs at admission SBP (mmHg)* SBP <100 mmHg Pulse rate (beats/min)* Pulse rate >120 beats/min RR (breaths/min)* RR >30 breaths/min SpO2 <90% Temperature <35°C or >38°C	140 (120–157) 31 (4.7) 90 (77-108) 68 (10.2) 20 (18-25) 42 (8.8) 132 (20.0) 72 (11.8)	18 18 18 18 206 206 24 76	135 (120–151) 24 (2.9) 88 (76-102) 62 (7.5) 20 (18-26) 84 (11.9) 104(12.7) 67(8.8)	23 23 20 20 144 144 31 84	132 (119–150) 61 (6.3) 90 (78-106) 94 (9.7) 20 (18-26) 90 (10.3) 141 (15.2) 56 (6.3)	23 23 24 24 120 120 62 95	0.001 0.003 0.021 0.130 0.357 0.221 0.001 0.001
Clinical signs at admission Rales Wheezing Rhonchi Confusion	314 (49.3) 145 (24.5) 171 (28.9) 49 (7.4)	47 92 93 20	342 (43.5) 221 (29.8) 180 (25.3) 49 (5.9)	63 108 138 17	385 (42.5) 264 (31.2) 195 (24.3) 64 (6.7)	84 144 188 30	0.021 0.018 0.132 0.511
*Data are presented as median (Q1-Q3) SBP: Systolic Blood Pressure; RR: Respiratory Rate; SpO2: Saturation Level of Oxygen by Pulse Oximetry.							

**Table 3:** Treatments in the emergency department and outcomes of patients.

	Winter (February)(N = 684)		Spring (May)(N = 849)		Autumn (October)(N = 991)		
	N (%)	MissingData	N (%)	Missing Data	N (%)	Missing Data	P-value
Treatment in the ED Oxygen Inhaled bronchodilator Antibiotic IV diuretics Systemic corticosteroid Anticoagulant IV vasodilators Non-invasive ventilation Antiplatelet agent Morphine Sublingual/oral vasodilators IV inotrope/vasopressor Mechanical ventilation	454 (69.9) 282 (42.5) 208 (31.6) 176 (27.1) 161 (24.5) 61 (9.5) 54 (8.4) 39 (6.0) 36 (5.6) 26 (4.0) 18 (2.8) 12 (1.9) 10 (1.5)	34 20 26 34 26 39 37 35 37 38 37 37 34	516 (62.9) 308 (37.8) 198 (24.4) 181 (22.3) 182 (22.2) 55 (6.8) 47 (5.9) 50 (4.6) 38 (4.7) 37 (4.6) 13 (1.6) 6 (0.8) 8 (1.0)	28 33 38 38 30 37 46 28 38 45 46 45 28	510 (53.4) 332 (34.9) 263 (27.7) 184 (19.5) 190 (20.1) 58 (6.1) 49 (5.2) 51 (5.3) 41 (4.4) 40 (4.2) 22 (2.3) 10 (1.1) 10 (1.1)	35 40 41 45 44 46 48 35 52 47 48 66 37	<0.001 0.009 0.009 0.002 0.109 0.035 0.033 0.757 0.539 0.858 0.311 0.142 0.558
Discharge from the ED To home To ward To intensive care unit Death in ED	253 (38.8) 328 (50.3) 63 (9.7) 8 (1.2)	32 32 32 32	306 (37.9) 442 (54.7) 54 (6.7) 6 (0.7)	41 41 41 41	362 (38.6) 505 (53.8) 63 (6.7) 9 (1.0)	52 52 52 52	0.929 0.261 0.047 0.638
In-hospital mortality	31 (7.9)	0	35 (7.1)	0	41 (7.2)	1	0.875
ED: Emergency Department; IV: Intravenous.							

### 3.3. Seasonal differences

The cohort included in the winter period population was significantly older compared to spring and autumn (P < 0.001). There was no significant difference between seasons in terms of sex and symptom duration (P = 0.119 and P = 0.627, respectively). The rate of ambulance arrival to ED was lower in the winter period as compared to spring and autumn (P < 0.001). The most common diagnoses were lower respiratory tract infections (LRTI), heart failure, COPD exacerbation, and asthma in all 3 seasons. The lower respiratory tract infection rate was higher in the winter period (35.1%), whereas the asthma rate was higher in the spring period (10.1%). The patients had, more often than not, a prior history of chronic heart failure, hypertension, diabetes, dyslipidemia, and obesity in the winter period (Table 1).

Vital signs at admission were more hypertensive, more hypoxic, and more hyper/hypothermic in the winter with less hypotension seen in the spring period compared to other seasons. For clinical signs at admission, rales were higher in winter, and wheezing was higher in spring (P < 0.05).

During initial ED care, about 2/3 of patients received oxygen therapy with a significantly higher proportion receiving it during winter. The other most common treatments given in ED were those involving an inhaled bronchodilator. About 1/4 of patients were given systemic corticosteroids that were not significantly differing among seasons. Patients more often received intravenous vasodilators and anticoagulant treatment during the winter period.

Although the rates of discharged home and admission to any ward did not differ between seasons, there was a significantly higher admission rate to ICU rate during the winter period. ED mortality was about 1% and, in hospital, mortality for admitted patients was 7.4%. Although both mortality rates seemed to be higher in the winter period, the results were not statistically significant.

## 4. Discussion

The present study explores the potential influence of seasons on epidemiology and contemporary management of dyspnea in EDs in Europe. Our results confirm that patients presenting to EDs with dyspnea were generally older patients with a median age of 69 years or older (median age of 72 years) during winter. As a part of physiologic aging, structural changes occur in the respiratory system and in the control of responses to hypoxia and hypercapnia. Dyspnea is very common in the elderly, with a prevalence ranging from 20%–38% in those over 65 years of age [9,10]. Cold weather can irritate airways by not only causing bronchial irritation and cough but also inducing bronchial tightening/constriction, which is more prominent in the elderly [11,12].

In our study, the most frequent comorbidities were cardiac and respiratory diseases. Dyspneic patients presented to EDs during the winter had more comorbidities, not only in terms of number but also in terms of diversity of comorbidities adding to cardiac/respiratory comorbidities, endocrinological comorbidities (such as diabetes, dyslipidemia, or obesity), and cognitive dysfunction. These concomitant illnesses posed a challenge for the ED management of dyspneic patients and increased ICU hospitalizations. Because breathlessness is accepted as a stronger predictor of 5-year survival than tests of pulmonary function and as they are also associated with ED reattendance and hospital admission [1], ED physicians should be more attentive to these patients, especially during peak periods in EDs such as in winter. These patients more often get systemic corticosteroids, which may complicate diabetes even more [3].

In this study, only the patient numbers recruited in winter are lower than for other seasons. This might be due to the diagnosis of asthma (which significantly increased in autumn and spring season). It is a relatively common diagnosis encountered in adult patients presenting with dyspnea to EDs [3,13–14]. Together with this, there is lower presentation of ED with ambulance in the winter season, and this might also have several causes. Ambulance to patient’s arrival time is usually delayed during the winter season [14], which may result in a relative increase in self-admittance to EDs. The other reason might be due to an increased referral of patients to ED by a family physician and/or general practitioners during winter seasons. In a study from Sweden, it was reported that patients were exposed to cold, ambient temperature in ambulances, and this is a common problem in prehospital care [15]. This might be another reason for higher self-referral in the winter season.

Seasonal variations, with increased morbidity in winter, have been shown in several clinical etiologies of dyspnea, as they have also shown for cardiovascular, pneumonia, and COPD exacerbations [16–19]. The most common causes of dyspnea in EDs were pulmonary infection, decompensated heart failure, and COPD exacerbations, which were significantly higher in winter. Our study demonstrates that although the etiology of dyspnea does not vary with seasons, their presenting condition is more serious in winter due to a significant increase in comorbidities and clinical signs suggesting a critical condition such as low oxygen saturation (20%) and hypo/hyperthermic instances (12%) on admission to ED. For ED management of dyspneic patient, patients received more supplemental oxygen, intravenous diuretics, vasodilators, antibiotics, anticoagulants, and inhaled bronchodilators during the winter season compared to other seasons. There is nearly two-fold increase in the admittance of dyspneic ED patients to intensive care units during winter. Our results seem to be in accordance with previous studies on specific etiologies of dyspnea as COPD exacerbations, asthma, lower respiratory tract infections, and heart failure were more often admitted in winter. A number of studies show that seasonal variations in dyspnea may be attributed, in part, to the fact that respiratory infections are more common during the winter months [1,3,17,20,21]. All of these findings show that dyspneic patients require immediate and high-quality clinical care, especially during winter.

Our study has several limitations. Since this was international multicenter registry, it should be taken into account that centers may have recorded data differently and there may be differences in health care access between countries that influence the usage of EDs. In addition, another main limitation of our study was that we did not include the summer season, which might have been very important for seasonal variation comparison. There was no central committee adjudicating the final diagnosis. However, local data collectors had the possibility to contact the coordinating center if they had any queries regarding data collection processes, therefore minimizing bias. There is also a modest amount of missing data for some cases, and this may also have influenced our results.

## 5. Conclusion

The present findings support the conclusion that dyspnea is a common cause of ED visits, especially during the winter season. During winter, dyspneic patients tend to be older and prefer to come themselves to the ED rather than with the help of an ambulance. They have significantly higher ED diagnoses of lower respiratory tract infection, heart failure, and COPD exacerbations. Admission to the ICU is also higher in winter. The analytic method and the outcome of this study may help guide practitioners in allocating ED resources more efficiently and in suggesting seasonal ED management protocols based on the seasonal trends of dyspneic patients.

## EURODEM Steering Committee:

Said Laribi (Chair, France), Mehmet Akif Karamercan (Turkey), Oene van Meer (the Netherlands), Richard Body (United Kingdom), Veli-Pekka Harjola (Finland), Adela Golea (Romania), Franck Verschuren (Belgium), Michael Christ (Germany), Cinzia Barletta (Italy), and Luis Garcia-Castrillo (Spain)

**EURODEM Study Group (includes all hospitals that provided data):**

**- France:** Patrick Plaisance, Ghanima Al Dandachi (CHU Lariboisière, Paris), Maxime Maignan (CHU, Grenoble), Dominique Pateron, Christelle Hermand (CHU, Saint Antoine, Paris), Cindy Tessier (CHU, Dijon), Pierre-Marie Roy (CHU, Angers), Lucie Bucco (CH, Chalon sur Saône), Nicolas Duytsche (CH, Macon).

**- The Netherlands:** Titus Schönberger (Jeroen Bosch Hospital, Hertogenbosch), Constant Coolsma (Medical Center Leeuwarden, Leeuwarden), Maarten Baggelaar (Canisius Wilhelmina Hospital, Nijmegen), Noortje Fransen (Elisabeth-TweeSteden, Tilburg), Crispijn van den Brand (Haaglanden Medical Center, the Hague), Doutsje Idzenga (Hospital St. Jansdal, Harderwijk), Maaike Maas (Catharina Hospital, Eindhoven), Myriam Franssen (Zuyderland, Heerlen), Charlotte Mackaij-Staal (St. Antonius Hospital, Nieuwegein), Lot Schutte (OLVG, Amsterdam), Marije de Kubber (Leiden University Medical Center, Leiden), Lisette Mignot-Evers (Máxima Medical Center, Eindhoven), Ursula Penninga-Puister (Wilhelmina Hospital, Assen), Joyce Jansen (Academic Medical Center, Amsterdam), Jeroen Kuijten (Elkerliek Hospital, Helmond), Marna Bouwhuis (Erasmus Medical Center, Rotterdam).

**- United Kingdom:** Richard Body (Manchester Royal Infirmary), Adam Reuben (Royal Devon and Exeter NHS Foundation Trust), Jason Smith (Plymouth Hospitals NHS Trust), Shammi Ramlakhan (Sheffield Teaching Hospitals), Melanie Darwent (Oxford Radcliffe Hospitals NHS Foundation Trust), James Gagg (Taunton and Somerset NHS Foundation Trust), Liza Keating (Royal Berkshire NHS Foundation Trust), Santosh Bongale (Inverclyde Hospital), Elaine Hardy (University Hospital, Birmingham), Jeff Keep (King’s College Hospital NHS Foundation Trust), Heather Jarman (St. George’s Healthcare NHS Trust), Steven Crane (York Teaching Hospital NHS Foundation Trust), Olakunle Lawal (Basildon and Thurrock), Taj Hassan (Leeds Teaching Hospitals NHS Foundation Trust), Alasdair Corfield (Royal Alexandra Hospital), Matthew Reed (Infirmary of Edinburgh).

**- Turkey:** Mehmet Akif Karamercan (Gazi University, Faculty of Medicine Hospital, Ankara), Mehmet Ergin (Necmettin Erbakan University, Meram Faculty of Medicine Hospital, Konya), Zerrin Defne Dündar (Necmettin Erbakan University, Meram Faculty of Medicine Hospital, Konya), Yusuf Ali Altuncı (Ege University, Faculty of Medicine Hospital, İzmir), Mücahit Avcil (Adnan Menderes University, Medical Faculty Hospital, Aydın), Yavuz Katırcı (Ankara Education and Research Hospital, Ankara), İbrahim Arzıman (Gulhane Military Medical Academy Hospital, Ankara).

**- Finland:** Hanna Suurmunne, Liisa Kokkonen (Päijät-Häme Social and Health Care Group, Lahti), Jukka Tolonen, Juha Valli (Helsinki and Uusimaa Hospital District, Hyvinkää), Minna Kiljunen (North Karelia Central Hospital and Honkalampi Centre, Joensuu), Jukka Tolonen (Helsinki University Hospital, Helsinki), Sanna Kaye (City of Helsinki Department of Social Services and Health Care, Helsinki), Jukka Tolonen, Mikko Mäkelä (Helsinki University Hospital, Espoo), Jukka Tolonen, Juhani Metsäniitty (Helsinki University Hospital, Vantaa), Eija Vaula (Satakunta Central Hospital, Pori).

**- Romania:** Sorana Truță (Emergency Department of the County Emergency Hospital, Târgu Mureș), Natalia Hrihorișan (Emergency Department of the County Emergency Hospital, Oradea), Diana Cimpoeșu (University of Medicine and Pharmacy, Emergency Department of the University County Emergency Hospital, Iași), Luciana Rotaru (University of Medicine and Pharmacy, Emergency Department of the University County Emergency Hospital, Craiova), Alina Petrică (Emergency Department of the County Emergency Hospital, Timișoara), Mariana Cojocaru (Emergency Department of the Emergency Hospital, Elias București), Silvia Nica (Emergency Department of the University Emergency Hospital, București), Rodica Tudoran (University of Medicine, Emergency Department of the County Emergency Hospital, Constanța), Cristina Vecerdi (Emergency Department of the Emergency Hospital, Brașov), Monica Puticiu (Emergency Department of the Emergency Hospital, Arad).

**- Belgium:** Alexandre Ghuysen (Centre Hospitalier Universitaire, Liège), Marc Vranckx (Centre Hospitalier Universitaire, Charleroi), Franck Verschuren (Cliniques Universitaires, Saint-Luc Brussels).

**- Germany:** Michael Christ, Felicitas Geier, Yvonne Smolarsky (Department of Emergency and Critical Care Medicine, Paracelsus Medical University, Nuremberg), Sabine Blaschke (Department of Emergency Care Medicine, University of Goettingen), Clemens Kill, Andreas Jerrentrup (Department of Emergency Care Medicine, University of Marburg), Christian Hohenstein (Department of Emergency Care Medicine, University of Jena), Felix Rockmann, Tanja Brünnler (Department of Emergency Care Medicine, Krankenhaus Barmherzige Brüder, Regensburg).

**- Italy:** Cinzia Barletta (St Eugenio Hospital, Rome), Giorgio Carbone (Gradenico Hospital, Turin), Roberto Cosentini (Polyclinic Hospital, Milan).

**- Spain:** Pablo Garmilla (Hospital Universitario Marques Valdecilla).
